# Soil organic matter stoichiometry as indicator for peatland degradation

**DOI:** 10.1038/s41598-020-64275-y

**Published:** 2020-05-06

**Authors:** Jens Leifeld, Kristy Klein, Chloé Wüst-Galley

**Affiliations:** Agroscope, Climate and Agriculture Group, Research Division Agroecology and Environment, Reckenholzstrasse 191, CH 8046 Zurich, Switzerland

**Keywords:** Biogeochemistry, Environmental sciences

## Abstract

Peatlands accumulate organic matter (OM) under anaerobic conditions. After drainage for forestry or agriculture, microbial respiration and peat oxidation induce OM losses and change the stoichiometry of the remaining organic material. Here, we (i) evaluate whether land use (cropland CL, grassland GL, forest FL, natural peatland NL) is associated with different peat stoichiometry, (ii) study how peat stoichiometry changes with OM content and (iii) infer the fate of nitrogen upon soil degradation. Organic C and soil N were measured for 1310 samples from 48 sites in Switzerland, and H and O for 1165. The soil OM content and C/N ratio were most sensitive to land use and are hence best suited as indicators for peatland degradation. OM contents (CL < GL < FL < NL), H/C, O/C, C/N ratios, and OM oxidation states were significantly different between land use types in top- and subsoils. With decreasing bulk OM content, C was relatively depleted while H and particularly N were higher. The data suggest very high N mobilization rates from strongly decomposed peat in agricultural topsoil. A comparison to peat C and N from mostly intact peatlands of the Northern hemisphere reveals that agriculture and, to a lesser extent, forestry induce a progressed state of soil degradation.

## Introduction

The formation and sequestration of soil organic matter (SOM) plays an important role in the terrestrial carbon (C) cycle and is considered an important building block for climate change mitigation strategies^1^. Organic matter in soils is mostly composed of C, hydrogen (H), oxygen (O) with additional contributions from nitrogen (N), phosphorus (P), and sulfur (S). These nutrients are considered ‘organic’ as they are mostly derived from organic molecules. Following microbial metabolism and transformation of litter, nutrient contents of SOM increase relative to C. Indeed, there is a strong negative but nonlinear relationship between organic SOC and the latter three major nutrients, i.e., SOM of mineral soils with low soil organic carbon (SOC) concentrations is relatively richer in N, P and S than soils containing more SOC^2^. The close link between SOC and organic nutrients has stimulated the hypothesis of co-sequestration of SOC and nutrients in mineral soil^3,4^ and, conversely, fostered suggestions that the net N release from mineral soil is inversely related to the soil’s C/N ratio^5^.

SOM stoichiometry is however less well studied in organic soils formed in peatlands. Together with SOC, intact peat-accumulating mires also sequester N and other nutrients^6^. C/N ratios of organic soils formed by peat accumulation are, however, much higher than those of mineral soils: A comprehensive survey of peatland sites in the northern hemisphere revealed a median C/N ratio of 49.0^7^, whereas the average C/N ratio of mineral soils worldwide is only 10.9^8^. This indicates a less pronounced microbial imprint on SOM stoichiometry of peat compared to mineral soil OM, in line with its often fibric or hemic appearance and substantial plant-derived contributions^9,10^.

Because peat is more strongly transformed by microbes the deeper and older the material is, decreasing C/N ratios with depth would be expected. This has been shown for some intact bogs^6,11,12^. However, evidence for such trends is conflicting^13–15^, probably because (i) changing vegetation communities, (ii) changing N deposition rates, or (iii) alternating dry and wet conditions with peat formation over time override a clear trend of C/N with increasing decomposition^10^. On the other hand, data from artificially drained and decomposed peat reveal low C/N ratios^16^, suggesting that with increased peat decomposition, N accumulates relative to C. However, this has not yet been studied along a series of peat materials from different sites and with different OM contents. Considering that organic soils drained for agriculture lose C at a faster rate than forests^17^ and that these soils also receive larger amounts of fertilizer, lower peat C/N ratios might be expected under agriculture.

Measurements of organically bound H and O in peat reveal a wide range of molar H/C and O/C ratios of 1.1–1.5 and 0.4–0.9, respectively^15,18^. During peat formation, H and O decrease relative to C, owing to the release of inorganic compounds with high O/C and H/C ratio, namely CO_2_ and CH_4_. Likewise, polysaccharides such as cellulose, which is relatively rich in O and C [(C_6_H_10_O_5_)n], degrade faster whereas aliphatic and aromatic compounds with lower O/C and H/C, either originating from the primary input material or newly formed during microbial transformation, accumulate^19,20^. These processes determine the resulting stoichiometry and oxidation state of SOC, which might be representative of the degree of peat decomposition^21^. In turn, peat composition may change again upon aerobic decomposition after drainage, but a systematic investigation of drainage and land use effects on stoichiometry is still outstanding.

Many organic soils in the temperate zone, particularly in Europe, have been subject to intensive drainage and land use change^22^, and it is known that this has caused soil subsidence and GHG emissions^23,24^. However, the land use impact on peat composition is less well known. Here, we study peat samples from managed and natural sites (Table 1), all from the cool temperate moist zone of Switzerland to infer, (i) whether land use influences the OM content and stoichiometry of peat, (ii) whether peat is composed differently in high and low OM content peats, and (iii) the possible fate of N upon peat degradation. The situation in Switzerland^25^ is similar to that of some other European countries in that many organic soils are already lost, and the remaining peatlands are mostly drained and managed^26^. Finally, a comparison to published C and N data from the Northern Hemisphere peatlands places the degree of peat degradation in a wider context.

**Table 1:** Site overview.

Site name	Coordinate WGS 84	Elevation (m asl)	Mean annual temperature (°C)	Mean annual precipitation (mm)	Soil pH (topsoil)^1^	Land use	Reference for site
Brüttelen	47.03°N, 7.18°E	438	9.9	1009	6.7	CL	a
Cressier_Mis.	47.04°N, 7.05°E	430	10.2	1066	7.9	CL	b
Gals I	47.04°N, 7.07°E	430	10.1	1073	6.5	CL	c
Im Moos	47.38°N, 9.57°E	414	10.1	1392	6.0	CL	a
Lüchingen	47.38°N, 9.57°E	414	10.1	1392	6.1	CL	a
Parzelle33	46.98°N, 7.05°E	431	10.2	981	6.6	CL	d
Spring	46.98°N, 7.05°E	431	10.2	981	5.5	CL	this study
Treiten	47.01°N, 7.15°E	439	10.0	1011	6.2	CL	a
Staatswald I	46.98°N, 7.09°E	431	10.1	994	6.3	CL	this study
Mühleturnen	46.82°N, 7.52°E	544	8.8	1083	6.3	CL	a
Ägeriried	47.07°N, 8.40°E	910	9.3	1343	3.4	FL	this study
Bannwald	47.01°N, 8.19°E	860	7.0	1527	3.6	FL	a
Birmensdorf	47.36°N, 8.45°E	560	9.3	1137	4.1	FL	a
Dévin des Dailles I	46.52°N, 6.96°E	1100	6.2	1470	3.9	FL	e
Dévin des Dailles II	46.52°N, 6.96°E	1100	6.2	1470	3.7	FL	e
Foremoos	47.01°N, 8.21°E	960	6.8	1653	3.8	FL	e
Gals II	47.04°N, 7.07°E	430	10.1	1073	7.3	FL	c
Hagenmoos	47.24°N, 8.52° E	600	9.0	1262	4.1	FL	this study
Joux Derrière	46.58°N, 7.00°E	1080	6.4	1331	3.8	FL	e
Maas	47.36°N, 8.46°E	560	9.0	1136	3.9	FL	e
Meiestossmoos I	47.01°N, 8.21°E	960	6.8	1653	3.9	FL	e
Meiestossmoos II	47.01°N, 8.21°E	960	6.8	1653	3.8	FL	e
Meiestossmoos III	47.01°N, 8.21°E	960	6.8	1653	3.6	FL	a
Sigigerwald	47.05°N. 8.15°E	830	7.7	1333	4.3	FL	e
Sömmerigchopf I	47.21°N, 9.40°E	1300	6.3	1971	4.5	FL	e
Sömmerigchopf II	47.21°N, 9.40°E	1300	6.3	1971	3.9	FL	e
Sömmerigchopf III	47.21°N, 9.40°E	1300	6.3	1971	4.8	FL	a
Staatswald II	46.98°N, 7.09°E	431	10.1	994	3.4	FL	this study
Staatswald III	46.98°N, 7.09°E	431	10.1	994	3.3	FL	this study
Staatswald IV	46.98°N, 7.09°E	431	10.1	994	4.2	FL	a
Vorderwengi I	47.20°N, 9.10°E	1070	6.3	2152	3.4	FL	a
Vorderwengi II	47.20°N, 9.10°E	1078	6.3	2152	4.1	FL	e
Weidli	46.77°N, 7.28°E	850	7.7	1262	3.9	FL	a
Chiemiwald	46.85°N, 7.17°E	570	9.1	1060	3.5	FL	e
Cressier II	47.04°N, 7.05°E	430	10.2	1066	7.4	GL	b
Eigenried	47.09° N, 8.53°E	985	9.1	1413	4.7	GL	this study
Gals III	47.04°N, 7.07°E	430	10.1	1073	6.3	GL	c
Kirchenthurnen	46.82°N, 7.52°E	540	8.8	1083	5.5	GL	a
Mühleturnen	46.82°N, 7.52°E	540	8.8	1083	6.0	GL	a
Rüthi	47.28°N, 9.54°E	435	10.2	1229	5.8	GL	a
Sömmerigchopf IV	47.22°N, 9.40°E	1300	5.4	2151	5.3	GL	a
Seebodenalp I	47.03° N, 8.27° E	1025	9.3	1171	3.6	GL	d
Seebodenalp II	47.03° N, 8.27° E	1025	9.3	1171	4.3	GL	this study
Seebodenalp III	47.05° N, 8.27° E	1077	9.3	1099	4.6	GL	this study
Vorderwengi III	47.20°N, 9.10°E	1070	6.3	2152	7.0	GL	a
Tourbière au Paquier dessus	46.67°N, 7.16°E	1440	5.2	1791	3.8	NL	e
Rüchiwald	46.88°N, 8.04°E	1640	4.8	2039	3.8	NL	e
Etang de la Gruyere	47.24° N, 7.05° E	998	6.5	1554	4.0	NL	this study
1 in water							

a^45^, b^46^, c^47^, d^19^, e^18^.

## Results

All measured chemical indicators are related significantly to land use (Tables 2 and 3). Contents of OM and SOC differed significantly between each land use type in the order CL < GL < FL < NL, both for the whole soil profile as well as the top- and subsoil samples individually. OM and SOC contents were higher in subsoils and, for whole profiles, above the average of all samples under FL and NL and below average for CL and GL. Peat C/N ratios also differed significantly between land use and increased together with OM contents. As for OM and SOC, values were above average for FL and NL. Nitrogen contents, for comparison, varied less and were slightly but significantly higher for peat only under GL. We also analysed six adjacent sites with different land use (Table 1, sites Cressier, Gals, Mühleturnen, Sömmerigchopf, Staatswald, Vorderwengi). The direct comparison of paired sites reveals that topsoil SOC contents were, on average, 0.1, 2.6, and 0.5% higher for GL vs. CL, FL vs. GL, and FL vs. CL. Soil C/N ratios were higher by 1.2, 2.9, and 8,7 units for the same pairs.

**Table 2:** Key chemical properties (mean ± one SE) of the studied peat samples, classified by land use. CL cropland, GL grassland, FL forest, NL natural land. H/C and O/C ratios are on a molar basis, C/N ratios on a per mass basis.

OM		SOC	N	H/C	O/C	C/N	C_ox_	OR
mg g^−1^								
all	802.9 (6.7)	423.4 (3.5)	17.8 (0.2)	1.37 (0.01)	0.55 (<0.01)	25.7 (0.3)	−0.15 (0.01)	1.07 (−0.01)
CL	569.7 (18.6)a	311.9 (9.4)a	17.2 (0.4)a	1.47 (0.02)c	0.54 (0.01)b	17.9 (0.4)a	−0.22 (0.03)a	1.10 (0.01)b
GL	668.9 (13.9)b	357.5 (7.8)b	18.4 (0.3)b	1.46 (0.01)c	0.58 (0.01)c	19.6 (0.4)b	−0.15 (0.02)b	1.07 (<0.01)a
FL	893.3 (4.7)c	476.1 (2.8)c	17.7 (0.2)a	1.30 (0.01)a	0.54 (<0.01)b	29.6 (0.4)c	−0.12 (0.01)b	1.05 (<0.01)a
NL	953.9 (3.2)d	521.8 (3.3)d	17.1 (0.5)a	1.35 (0.01)b	0.52 (0.01)a	33.8 (1.1)d	−0.23 (0.01)a	1.08 (<0.01)b
**0–0.3 m**								
CL	451.4 (22.4)a	230.6 (10.7)a	15.5 (0.5)a	1.53 (0.03)c	0.56 (0.02)a	14.5 (0.3)a	−0.23 (0.05)a	1.10 (0.01)b
GL	573.7 (16.9)b	290.1 (9.7)b	18.1 (0.5)b	1.54 (0.02)c	0.63 (0.01)b	16.3 (0.5)b	−0.10 (0.03)b	1.07 (0.01)a
FL	878.8 (7.3)c	459.8 (3.7)c	18.8 (0.3)b	1.32 (0.01)a	0.57 (0.01)a	26.9 (0.6)c	−0.08 (0.01)b	1.05 (<0.01)a
NL	935.4 (5.1)d	483.7 (4.2)d	15.9 (0.9)a	1.39 (0.01)b	0.59 (0.01)a	35.7 (2.8)d	−0.12 (0.02)b	1.05 (<0.01)a
**>0.3 m**								
CL	682.9 (22.4)a	373.3 (11.2)a	18.5 (0.5)b	1.39 (0.02)c	0.52 (0.01)b	20.4 (0.5)a	−0.22 (0.02)b	1.09 (0.01)b
GL	785.4 (17.5)b	424.7 (9.8)b	18.8 (0.4)b	1.38 (0.01)c	0.53 (0.01)b	23.0 (0.5)b	−0.20 (0.02)b	1.08 (0.01)b
FL	904.8 (6.1)c	488.9 (4.0)c	16.9 (0.3)a	1.29 (0.01)a	0.52 (0.01)b	31.7 (0.6)c	−0.15 (0.01)c	1.06 (<0.01)a
NL	963.1 (3.7)d	540.8 (2.6)d	17.6 (0.6)a	1.33 (0.01)b	0.48 (<0.01)a	32.9 (1.0)c	−0.29 (0.01)a	1.09 (<0.01)c

Number (n) of all samples for SOC, N and C/N: 1310, for OM, H/C, O/C: 1166, Cox and OR: 1156; n 0–0.3 m for SOC and C/N: 581, for OM, H/C, O/C: 531, Cox and OR: 528. Cox: degree of oxidation of organic C; OR: oxidative ratio according to 28, see methods.

Letters indicate differences in values between the different land use types (LSD, p < 0.05).

**Table 3:** ANOVA results for all soil samples and differentiated by top- and subsoil (0–0.3 and >0.3 m).

	OM			SOC			N			C/N			H/C			O/C			C_ox_		
	d.f.	F	p	d.f.	F	p	d.f.	F	p	d.f.	F	p	d.f.	F	p	d.f.	F	p	d.f.	F	p
**All samples**																					
Land-use	4	278.05	*	4	289.66	*	4	304.39	*	4	150.64	*	4	1748.1	*	4	568.54	*	4	14.96	*
Site	36	44.09	*	44	34.03	*	44	15.75	*	44	19.30	*	36	14.516	*	36	14.96	*	36	9.71	*
Error	1118			1261			1262			1262			1126			1115			1116		
**0–0.3 m**																					
Land-use	4	238.13	*	4	233.25	*	4	239.79	*	4	100.20	*	4	1294.1	*	4	458.42	*	4	5.37	<0.01
Site	35	43.14	*	44	43.13	*	35	22.84	*	44	15.62	*	35	12.39	*	35	9.92	*	35	5.46	*
Error	487			533			502			533			490			486			487		
**>0.3 m**																					
Land-use	4	397.57	*	4	307.24	*	4	301.9	*	4	160.25	*	4	1722.5	*	4	574.71	*	4	28.24	*
Site	33	23.38	*	33	21.48	*	33	8.01	*	42	12.05	*	33	9.26	*	33	10.90	*	33	7.24	*
Error	590			621			620			683			597			590			590		

^*^P < 0.001.

Ratios of H/C were the highest under CL and GL, whereas O/C ratios were the highest under GL only. In all four land use situations, H/C and O/C were higher in top- than in subsoils. Oxidation states of OM also differed between land use types, but in contrast to the parameters above, intensive land use (CL and GL) did not lead to consistently different C oxidation state (C_ox_) values (Table 2). Across all sites, C_ox_ was higher in topsoil than in subsoil.

**Figure 1:**
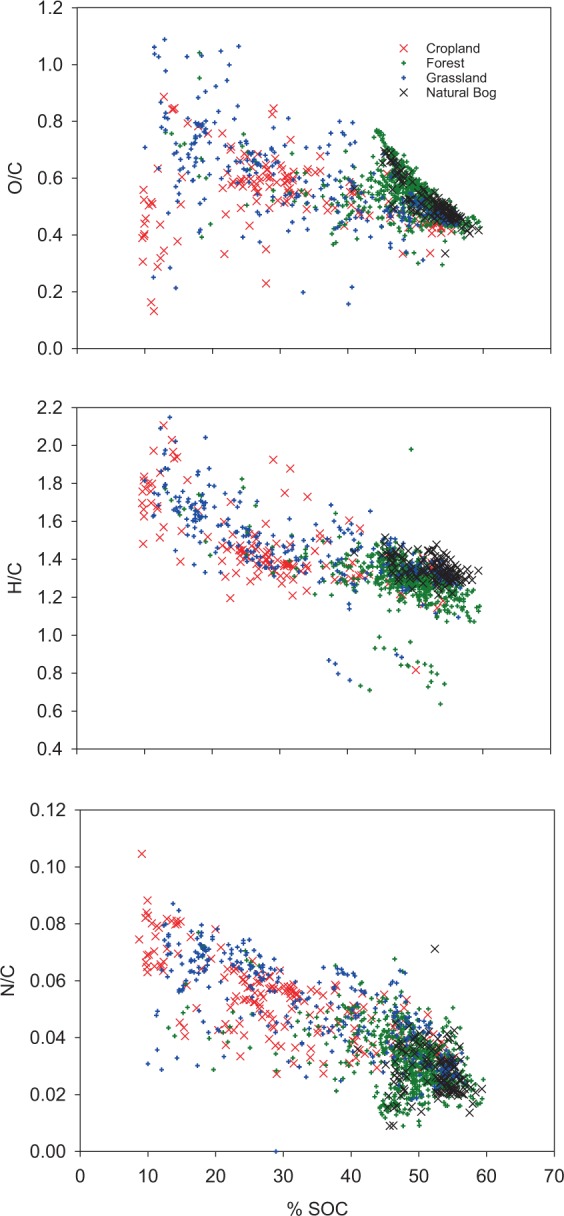
Soil organic matter O/C (upper panel), H/C (middle), and N/C (lower panel) ratios as related to SOC concentration in peatlands. Symbols represent the four studied land use types. Coefficients of determination for linear regressions were 0.22, 0.50, and 0.59 for O/C, H/C, and N/C, respectively (For details, see Suppelementary Table S1).

**Figure 2:**
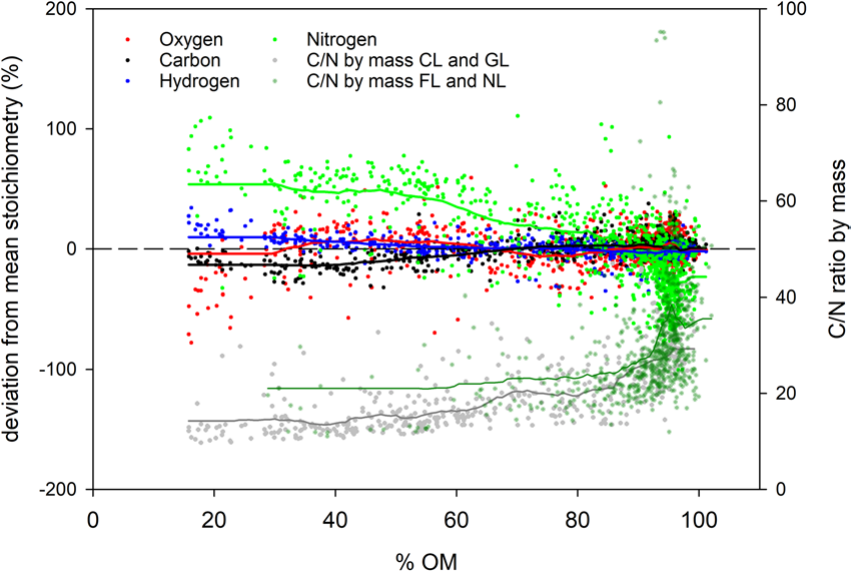
Deviation in sample stoichiometry from the average stoichiometry, based on molar percentages, of the whole data set for each element. In addition, soil C/N ratios are shown. Data are plotted as a function of soil OM content. Lines represent running averages (n = 10 samples) for each parameter. For C/N ratios, the running average for samples from forest and natural sites is displayed as a solid dark green line, and the one for samples from cropland and grassland sites as a solid grey line.

Molar ratios were inversely and significantly related to SOC content. The strengths of the relationships increased in the order O/C < H/C < N/C (Fig. 1). Additionally, there were interactions between OM content and OM stoichiometry (Fig. 2). Linear regression of data in Fig. 2 indicates that, for a one percent reduction in OM content, the relative (molar) contributions of N and H to OM increase by 1.28 and 0.18%, respectively; whereas the relative contributions of C and O decrease by 0.22 and 0.07%, respectively. All regression coefficients as related to Figs. 1 and 2 can be found in Supplementary Table S1.

To evaluate whether changes in soil C/N ratio with OM content differed between unfertilized (i.e., forest and natural sites) and fertilized (i.e., croplands and grasslands) sites, we fitted exponential functions through the corresponding subsets (please note that in Fig. 2, running averages are shown as a guide for the eye, not the curve fits). Results indicate (see Supplementary Table S2), that for both land use pairs, C/N ratios significantly drop with declining OM content. Further, the parameter estimates indicate that changes in C/N occur at a higher rate in forests and natural lands than in agricultural land above c. 75% OM, but at a similar rate <75% OM.

**Figure 3:**
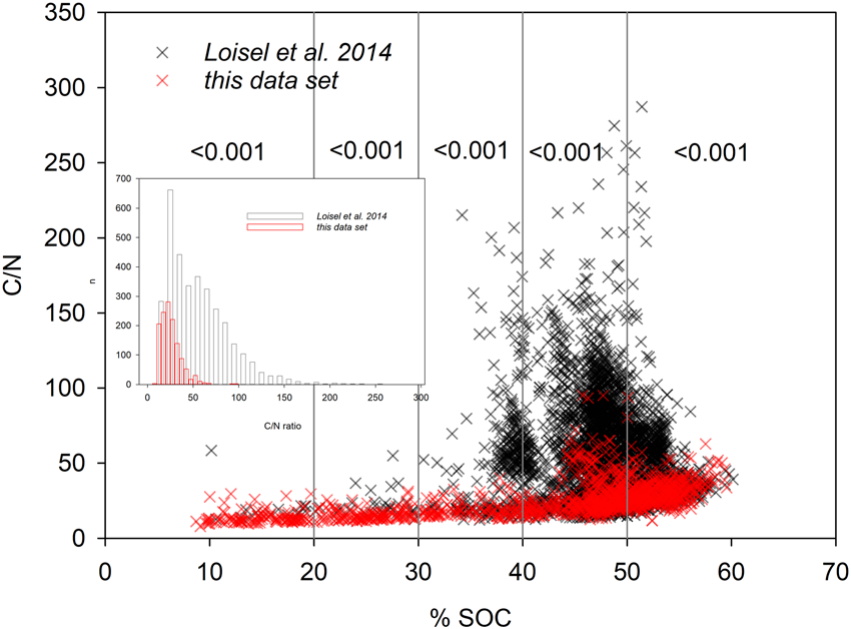
Comparison of C/N ratios and SOC contents of Northern Hemisphere peatlands^7^ with the current data set from temperate, mostly managed peatlands in Switzerland. Vertical lines group the data set into classes of different SOC content; error probabilities indicate whether C/N ratios of the current data set are significantly below that of the Loisel data set^7^. The small insert shows distribution of C/N ratios for both data sets.

Compared to the Northern Hemisphere data set^7^ which comprises mostly intact mires, our peat contained, on average, lower C (42.5 ± 0.3% vs. 46.9 ± 0.1%) but higher N (1.78 ± 0.01% vs. 1.19 ± 0.01%) content. Across the whole spectrum of C contents, C/N ratios of the current data set (mean 25.7 ± 0.3, median 23.7) were highly significantly below those of the Northern data set (mean 55.7 ± 0.6, median 48.7) (Fig. 3).

## Discussion

There is a clear and systematic difference in peat OM content and stoichiometry in organic soils between the four studied land use types in Switzerland. We presume that differences in OM content among land use types, as found here, mostly reflect drainage and land use effects, and are the result of long-lasting decomposition. Yet, it must be considered that land use is not evenly distributed across the landscape, with sites chosen in part depending on their climatic or topographic suitability, and that this might be linked to initial peat conditions. This was addressed by using a nested ANOVA approach and, where possible, a comparison of paired sites. The latter confirmed that land use exerts direct control on SOC, OM content, and C/N; and that these differences cannot be explained solely by preferential selection of sites for particular land use.

Soil OM concentration gradients are used here to exemplify how stoichiometry changes with peat degradation, as no real time-series is available. Although the initial OM content is not known and may vary between sites, we presume that samples containing >95% OM contents are representative of an undisturbed situation. Relative to this sample set (>95% OM, n = 325, of which 225 = FL, 57 = NL, 43 = GL, 0 = CL), the other peat samples contained on average 5.0 ± 0.5 (NL), 11.3 ± 1.3 (FL), 34.8 ± 1.4 (GL) and 40.0 ± 1.9 (CL) % less OM. Lower OM contents in more intensively managed and more deeply drained soils (CL and GL in our case), are in line with the order of CO_2_ flux measurements over drained peatlands under different land use^17^. The reported differences in OM contents are upper-end approximations, as peat layers of some sites (particularly fens) may have had OM contents lower than 95% in their pristine state. On the other hand, for some organic soils, losses might have been even greater: While this study examined only existing organic soils, (near-) complete losses of OM results in classification as a mineral soil (organic horizon less than 10 cm thick or with less than 20–30% OM depending on clay content and water saturation); which, in Switzerland, may extend over larger areas than still-existing peatlands^25^.

The C_ox_ integrates the stoichiometry into a single number. Across all samples, C_ox_ is more negative (i.e., C is more reduced) compared to that of to various plant litter types^27^, most likely as a consequence of anaerobic decomposition during peat formation. Accordingly, i) OM from intact peatlands show the lowest C_ox_; and ii) C_ox_ values tend to be more negative in subsoils, in agreement with other studies^21,28^. Elemental data were also converted to oxidative ratios (ORs), which are linearly and inversely related to C_ox_. Comparison with two further studies that report OR values for peatlands suggests that the peat described in this study is similar in terms of oxidation to peat reported elsewhere, but is much more variable. A study from Moor House, a blanket bog in northern England comprising different management practices, reported a range of OR values of 0.92–1.08 for 60 samples from the bog, and a greater range of 0.92–1.11 (median for Histosols 1.03) when literature values were included^28^. A study of 300 Canadian peatlands^21^ reported OR values of 1.00–1.18. Our OR values (median 1.07, range 0.81–1.43) encompass the OR values from these two studies and span a wider range, possibly because our sites represent a broader range of peatland ecosystems, and in contrast to the sites from^21^, also include drained and fertilised sites. The C in the peat samples from the current study is, on average, slightly less oxidized than that of Worrall, *et al*.^28^. This difference might be related to the type of peat sampled. Particularly, our analysis encompassed more strongly reduced peat from deeper layers (max. 2 m), whereas peat samples collected in^28^ were obtained only to 30 cm depth. Furthermore, because of ongoing subsidence of drained peatlands, a contemporary sampling depth of, for example, 1 m, may represent a soil layer at a depth of 3 m before drainage onset^29^, exposing older peat which is more aromatic and, consequently, has a higher OR, to the surface^30^.

Molar ratios of H, O, and N to C were higher for samples containing little SOC, suggesting a greater number of functional groups and, in case of H/C and N/C, a greater microbial imprint. There were a few samples with H/C ratios of <1 (Fig. 1). Inspection of these data revealed that they belong to six different sites, representing three land use types. In a data set from Ontario, Canada^21^, measured H/C ratios as low as 0.7 in their (peat accumulating) peatlands. We therefore presume that these ‘outliers’ may represent background variability. Elements H and particularly N were enriched in samples containing less OM in our study. The observed significant shift in stoichiometry along SOC and OM concentration gradients suggests that the molecular composition of degrading peat is systematically altered towards a higher share of N and H-containing moieties with ongoing decomposition. Importantly, we see this trend not only for the bulk data set, but also when forest and natural sites, that do not receive N fertilization, are studied separately (see running averages for C/N ratios in Fig. 2, and data in Supplementary Table S2). Hence, the enrichment of N over C with declining OM content cannot be attributed solely to N fertilization, but is also caused by a faster loss of C relative to N when peat degrades. A relative enrichment of N over C, or, in other words, a greater loss of C than N, has been reported for the top layers of extensively used, unfertilized drained grasslands elsewhere^13,16^. Also, these studies indicated that the process of peat decomposition itself, without further N input, reduces C/N ratios substantially. At the same time, declining C/N ratios are also caused by N fertilization in agricultural soils, particularly when cropped. Consequently, C/N ratios were the smallest in topsoils under agriculture (Table 2) and similar to C/N ratios found in drained fens in North Germany^31^.

Different land uses resulted in significant differences in OM stoichiometry in both top- and subsoil (Table 2). However, effects were less pronounced in subsoil. For all of the measured parameters, differences between maximum and minimum values were smaller in subsoils than in the upper 30 cm, indicating that land use change alters chemical peat composition more in upper, typically permanently drained layers. Comparison of H/C ratios of peat from this study with values from peatlands around the world^21^ illustrates this: Whereas the H/C ratios of deeper peat (>0.3 m) in this study are within the range of that reported from Canadian, UK and Latvian peatlands, the H/C ratios from shallower peat of CL and GL sites (from this study) are outside of the range reported for these other peats. Such high H/C ratios indicate high lipid content^32^, possibly related to increased microbial residues indicating high microbial transformation of peat. Additionally, soil C/N ratios were smaller in topsoils of managed systems, suggesting higher N input and/or advanced microbial transformation of the peat^33^. Notably, this result was reversed for the natural sites. Here, in accordance with other natural mire profiles^6,11^, a smaller C/N ratio in subsoil is maintained. Taken together, a reversed trend of C/N ratios with depth, as found for CL, GL, and FL, might be indicative of advanced peat decomposition in organic soils.

Net N release from mineral soil is expected to depend on the soil’s C/N ratio. For mineral soils, a high C/N ratio is associated with lower N release, whereas at ratios below 15, N release increases exponentially^5^. Mechanistically, the negative relationship between soil C/N and N release is explained by the difference in stoichiometry of the decomposing microbial biomass vs. the nutrient-poor plant litter material. With continuing microbial transformation, soil’s C/N ratio declines, approaching values of 10 and below^34^. A non-linear relationship has been also reported for the dependency of N_2_O emissions on C/N in organic soil: at C/N ratios of below 15, N_2_O emissions tend to increase sharply^35–37^. These studies indicate that even strongly degraded and managed organic soils bear the risk of high N losses, in line with constantly high OM mineralization rates also found in strongly degraded peat^38,39^. Our sites represent different stages of soil degradation, and as such indicate that with ongoing OM loss, N-release rates increase in conjunction with a decline in soil C/N. This occurs in spite of an accumulation of N relative to the other elements. Figure 2 and Table S1 indicate that the smaller the C/N ratio becomes, the more C and N are lost in tandem. The C/N ratios change only little (CL, GL) or or almost not at all (FL, NL) below 75% OM. At higher OM contents, N seems mostly retained within the soil system. Data in Table 2 allow an estimate of the gross N mobilization in organic soil. With an annual C loss rate of 8 t ha^−1^, a value typical for drained and intensively managed temperate peatlands^17^, topsoil of a cropland with an average C/N ratio of 14.5 may annually release c. 550 kg N ha^−1^, a magnitude in line with experimental evidence^40^. Although this calculation is only a rough estimate, it stresses that more attention should be paid to the N cycle and N loss in degrading peatlands.

Set into a wider geographical context, our peat samples contained less C and considerably more N, resulting in a different distribution of C/N ratios compared to the northern hemisphere data set of^7^. As discussed above, the relative enrichment of N over other elements in degrading peat as caused by the different stoichiometry of plant material and decomposer organisms may be one reason for this, considering that the sites in^7^ are mostly unmanaged and, hence, accumulate peat. In addition, intensively managed organic soils are fertilized and prone to substantial atmospheric N deposition. In the Swiss Central Plateau, where most of the studied cropland and grassland sites are situated, annual N deposition can reach 30–40 kg N ha^−1^, and even in peatland ecosystems and forests, N deposition records in Switzerland are 10–20 kg N ha^−1^a^−1^ and up to 50 kg N ha^−1^a^−1^ in single fens^41^. Nitrogen input into peatlands, particularly bogs, alters vegetation communities, increases peat mineralization, and impairs CO_2_ uptake^42–44^. Thus, with ongoing peat decomposition, the observed relative accrual of soil N and the postulated increase in N release may induce a positive feedback mechanism, where peat communities altered by N deposition are subjected to accelerated decomposition processes, thereby mineralizing more C and further exacerbating declining C/N ratios. Notably, C/N ratios in^7^ also exceeded our measurements for samples with >50% SOC. These C-rich samples were mostly from natural and forest sites, suggesting that external N input may also play a relevant role when peatlands are not managed.

## Methods

We took peat samples at 48 sites in Switzerland (Table 1) down to a maximum depth of 1–2 m. When peat thickness was less, samples were collected down to the underlying sediment or bedrock. Samples were either collected with a russian peat auger (Eijkelkamp, The Netherlands) or, when soils were more compacted (as is typically the case for agricultural sites), with a motor driven auger (Humax, Switzerland). Peat samples were taken in triplicate per site 25–50 m apart. Cores were cut into 3–10 cm increments, dried at 105 °C, milled, and measured for C, H, and N by dry combustion and elemental analysis, and for organic O after pyrolysis at 1000 °C and subsequent GC-TCD quantification (Hekatech, Germany). A few samples contained carbonate and were HCl fumigated before elemental analysis. Soil horizons in fens that showed clear signs of sediment layers were excluded from the analysis. In addition, a lower threshold of 10% SOC was used to distinguish peat from non-peat. This threshold does not compromise the minimum requirement for organic horizons defined by^17^, as samples with SOC contents between 10–20% were always part of a thicker organic horizon with higher SOC contents in other layers. Minimum thicknesses of organic horizons in sensu^17^ were 0.4 m. We assigned sites to land use by site inspection of the vegetation and sampled 10 CL sites, 11 GL sites, 24 FL sites, and 3 natural undisturbed bogs (NL). All of the cropland and grassland sites, and most of the forest sites were drained. In most cases, drainage commenced many decades to >one century ago. The average site altitude (m asl.) was 440 (CL), 805 (GL), 860 (FL) m and 1359 m (NL). Correspondingly, climate data across land-use types differed (see Table 1). Average mean annual temperatures were 10.0, 8.7, 7.7, and 5.5 °C and mean annual precipitation 1098, 1336, 1467, and 1795 mm for CL, GL, FL, and NL, respectively.

The number of samples from the four land use types are 193 (CL), 338 (GL), 662 (FL), and 117 (NL). Mean depths of all samples taken within one land-use were 0.45, 0.48, 0.44 and 0.70 m for CL, GL, FL, and NL, and mean maximum depth of sampling were 0.95, 0.75, 0.91, and 1.23 m for CL, GL, FL, and NL.

In total, 1310 samples were analysed for their SOC and N content, and 1165 for their O and H content. We refer to the sum of these four elements as soil organic matter. OM oxidation state (C_ox_) and oxidative ratios were calculated according to^27^. Effects of land use on OM content, H/C, O/C, C/N ratios, and C_ox_ were analysed using ANOVA with land-use as fixed factor and site as random factor nested in land-use to take account of possible differences in peat properties induced by preferential use of sites for specific land use. Differences between land-use was tested post-hoc by Fisher’s LSD test. The relationship between % OM and deviations in OM stoichiometry from the average OM composition, and the relationship between % SOC and molar ratios H/C, O/C, N/C were studied by linear regression. Peat C and N contents of our data set were compared with a much larger data set of mostly undisturbed peatlands from the Northern hemisphere published by^7^, and C/N ratios of the Northern data set and our data were compared for different classes of SOC content using a Mann-Whitney U-test.

## Supplementary information

Supplementary information.

Supplementary Tables.
