# Publisher Correction: Soil organic matter stoichiometry as indicator for peatland degradation

**DOI:** 10.1038/s41598-020-68763-z

**Published:** 2020-07-22

**Authors:** Jens Leifeld, Kristy Klein, Chloé Wüst-Galley

**Affiliations:** Agroscope, Climate and Agriculture Group, Research Division Agroecology and Environment, Reckenholzstrasse 191, CH 8046 Zurich, Switzerland

Correction to: *Scientific Reports* https://doi.org/10.1038/s41598-020-64275-y, published online 06 May 2020

A supplementary file containing Tables S1 and S2 was omitted from the original version of this Article. This has been corrected in the HTML version of the Article; the PDF version was correct at time of publication.

