# Supplementary material for: Soil organic matter stoichiometry as indicator for peatland degradation
Source: Sci Rep. 2020 May 6;10:7634. doi: 10.1038/s41598-020-64275-y (PMC7203203; doi:10.1038/s41598-020-64275-y)
Supplement: Supplementary file 2 — Supplementary Tables. [file 41598_2020_64275_MOESM2_ESM.pdf]

**Supplementary Material to: Soil organic matter stoichiometry as indicator for peatland degradation**

Jens Leifeld[1]*, Kristy Klein[1], Chloé Wüst-Galley[1]

[1]Agroscope, Climate and Agriculture Group, Research Division Agroecology and Environment, Reckenholzstrasse 191, CH 8046 Zurich, Switzerland

correspondence: Jens Leifeld
e-mail: jens.leifeld@agroscope.admin.ch
tel: +41 58 468 7510
orcid: 0000-0002-7245-9852

Table S1. Regression coefficients and their uncertainties for the dependency of SOM stoichiometry on SOC and SOM content (linear model, f(x) = a+b*x) for data shown in Figs. 1 and 2. Number of stars indicate error probability (* <0.05; ** <0.01; ***<0.001). Values in parenthesis are one standard error.

| | a | b | R2 |
|---|---|---|---|
| O/C vs. SOC (Fig. 1) | 0.7377 (0.0108)*** | -0.0043 (<0.001)*** | 0.2196 |
| H/C vs. SOC (Fig. 1) | 1.8024 (0.0134)*** | -0.0101 (0.003)*** | 0.4965 |
| N/C vs. SOC (Fig. 1) | 0.0805 (0.0010)*** | -0.0010 (<0.001)*** | 0.5883 |
| rel. H (Fig. 2) | 14.11 (1.07)* | -0.179 (0.013)** | 0.1746 |
| rel. O (Fig. 2) | -5.17 (2.11)*** | 0.069 (0.025)*** | 0.0081 |
| rel. C (Fig. 2) | -18.24 (1.15)*** | 0.217 (0.039)*** | 0.2105 |
| rel. N (Fig. 2) | 104.53 (3.37)*** | -1.282 (0.041)*** | 0.5227 |

Table S2. Regression coefficients for single exponential relationship between % OM and C/N ratio by mass according to f(x) = y0+a*exp(b*x). Number of stars indicate error probability (* <0.05; ** <0.01; ***<0.001; n.s. not significant). Values in parenthesis are one standard error. CL cropland, GL grassland, FL forest, NL natural peatland.

| | y0 | a | b | R2 |
|---|---|---|---|---|
| all samples | 14.22 (0.81)*** | 0.0842 (0.0545) n.s. | 0.0566 (0.0064)*** | 0.4102 |
| CL and GL | 13.17 (0.77)*** | 0.2367 (0.1543) n.s. | 0.0435 (0.0065)*** | 0.5428 |
| FL and NL | 17.43 (2.19)*** | 0.0286 (0.0441) n.s. | 0.0664 (0.0149)*** | 0.1908 |
